# A Coarse-to-Fine Feature Aggregation Neural Network with a Boundary-Aware Module for Accurate Food Recognition

**DOI:** 10.3390/foods14030383

**Published:** 2025-01-24

**Authors:** Shuang Liang, Yu Gu

**Affiliations:** 1 School of Biomedical Engineering, Capital Medical University, Beijing 100069, China; shliang@ccmu.edu.cn; 2 Laboratory for Clinical Medicine, Capital Medical University, Beijing 100069, China; 3 Beijing Key Laboratory of Fundamicationental Research on Biomechanics in Clinical Application, Capital Medical University, Beijing 100069, China

**Keywords:** food recognition, domain shift, deep learning, dietary management

## Abstract

Food recognition from images is crucial for dietary management, enabling applications like automated meal tracking and personalized nutrition planning. However, challenges such as background noise disrupting intra-class consistency, inter-class distinction, and domain shifts due to variations in capture angles, lighting, and image resolution persist. This study proposes a multi-stage convolutional neural network-based framework incorporating a boundary-aware module (BAM) for boundary region perception, deformable ROI pooling (DRP) for spatial feature refinement, a transformer encoder for capturing global contextual relationships, and a NetRVLAD module for robust feature aggregation. The framework achieved state-of-the-art performance on three benchmark datasets, with Top-1 accuracies of 99.80% on the Food-5k dataset, 99.17% on the Food-101 dataset, and 85.87% on the Food-2k dataset, significantly outperforming existing methods. This framework holds promise as a foundational tool for intelligent dietary management, offering robust and accurate solutions for real-world applications.

## 1. Introduction

Food recognition, typically referring to the classification of food types through image analysis, is a foundational task in food computing [1]. It plays a significant role in promoting healthy dietary management by helping individuals monitor their nutrient intake, avoid allergenic ingredients, and prevent diet-related health conditions such as hypertension, diabetes, heart disease, stroke, and cancer [2,3,4]. According to the World Health Organization (WHO), maintaining a healthy diet is fundamental to overall health, well-being, and optimal growth and development [5]. However, the significance of maintaining healthy diets is frequently underestimated, as most individuals lack proactive awareness and professional knowledge about nutrient intake and diet-related diseases [6]. Additionally, access to professional dietary guidance, such as from nutritionists, remains limited. This is particularly true in low- and middle-income countries, as well as in regions experiencing high levels of food insecurity [7].

With the rapid advancement of artificial intelligence, automatic food recognition leveraging deep learning (DL) techniques has achieved remarkable progress in various applications, such as dietary assessment, health monitoring, and mobile food-tracking applications [8,9,10]. Bossard et al. developed a large-scale image dataset comprising 101 food categories (named Food-101) for food recognition tasks and proposed an AlexNet [11]-based network, achieving a Top-1 accuracy of 56.4% [12]. The Food-101 dataset has been widely used and has become an important benchmark for evaluating food recognition tasks. VijayaKumari et al. proposed an Efficientnet-b0-based transfer learning framework, achieving a Top-1 accuracy of 80% [13]. Hamid et al. proposed a deep convolutional neural network with 54 layers to capture more complex feature representations, achieving a Top-1 accuracy of 88.28% [14]. Deng et al. proposed an attention-guided ConvNeXt-based framework [15] focusing on salient features specific to food items, achieving a Top-1 accuracy of 91.12% [16].

Despite the explorations and advancements achieved by these methods in food recognition, their performance remains limited, with challenges such as distinguishing subtle differences between food items and the insufficient scale and diversity of existing datasets, hindering further progress [17,18]. However, in our view, existing methods primarily focus on enhancing the backbone network by increasing its depth or introducing complex modules to improve feature representation capabilities. While these approaches have achieved certain performance gains, they fail to address the challenge of distinguishing subtle differences between food items.

We believe this issue is twofold: (1) The location information of food within an image is critical, as background elements can introduce noise. If a model places undue focus on background regions, it may struggle with intra-class consistency, leading to instability during learning, or confuse inter-class differences when items share similar backgrounds. (2) Existing food recognition datasets are usually collected from the internet and annotated manually. These datasets often exhibit significant variations in capture angles, lighting conditions, and devices, leading to notable differences in perspective, color distribution, and image resolution for the same food item. Such discrepancies amplify intra-class variability and domain-shift challenges.

To address these challenges, this paper presents a modular, multi-stage deep learning framework for food recognition named CBDTN. The proposed framework consists of three main components, i.e., the ConvNeXt-based backbone network, the boundary-aware module (BAM) and deformable ROI pooling (DRP), and the transformer encoder and the NetRVLAD module [19], with the following main contributions:

1. A boundary-aware module is proposed to enhance edge perception and is paired with a spatial attention mechanism to refine global features. By employing deformable ROI pooling to extract multi-regional local features from the upsampled global features, it constructs a unified global-local feature space that effectively captures both fine-grained details and contextual information.
2. A transformer encoder is employed to capture and integrate global and local features, followed by a NetRVLAD module for extracting relationships and aggregating features from them. This design ensures rotation-invariant features, reduces intra-class variations and domain shifts, and enhances the consistency and reliability of food recognition.
3. The proposed CBDTN framework achieves excellent accuracy and generalization, achieving Top-1 accuracies of 99.80%, 99.17%, and 85.87% on three public food recognition benchmarks. The three datasets, which consist of 2, 101, and 2000 food categories with sizes of 5 K, 100 K, and 1 M images, respectively, highlight CBDTN’s superior cross-domain performance and its potential as a robust tool for food recognition in intelligent dietary management systems.

The rest of this paper consists of five parts. Section 2 discusses related works. Section 3 describes the materials and methods. Section 4 discusses the results. Section 5 presents a discussion. Section 6 presents the conclusions.

## 2. Related Works

### 2.1. Food Recognition

Food recognition has garnered significant attention in recent years due to its broad applications in dietary management, health monitoring, and personalized nutrition [20]. As a foundational task in computer vision, food recognition presents unique challenges related to intra-class variability (e.g., different food preparations) and inter-class similarity (e.g., visually similar dishes). These challenges have been extensively addressed using deep learning-based methods, many of which have been evaluated on public datasets containing a diverse range of food categories [21,22,23].

Several methods have leveraged deep learning to improve food recognition accuracy. Gao et al. incorporated data augmentation and feature enhancement into vision transformers [24] to enhance the model’s global feature representation capability, achieving a Top-1 accuracy of 95.17% on standard datasets [25]. Similarly, Sheng et al. focused on the trade-off between performance and efficiency, proposing a lightweight vision transformer-based hybrid network that achieved a Top-1 accuracy of 90.7% [26]. These approaches have made notable strides but often require either high computational resources or compromise efficiency for better performance.

Early approaches primarily focused on small-scale datasets, achieving limited generalization across different food categories. Recent advances have been fueled by the introduction of larger and more diverse datasets, such as Food-2K [18], which stands out as the largest food dataset to date and is often referred to as the “ImageNet” of food recognition. The availability of such large-scale datasets has opened up new opportunities but also introduced significant challenges. One of the main challenges is the computational complexity and memory requirements for training models on such extensive datasets. Moreover, the wide variety of food items and their presentation styles (e.g., different cooking methods and cultural variations) present difficulties in achieving robust and generalized recognition. In our study, we take advantage of the larger, more challenging datasets, including Food-2K, to explore the scalability and cross-domain generalization of food recognition models. The comprehensive evaluation of datasets of varying scales highlights the versatility and scalability of our method, providing a solid foundation for tackling the challenges posed by large-scale food recognition tasks.

### 2.2. Network Architecture

The development of convolutional neural networks (CNNs) has significantly advanced image analysis, driving major improvements across a wide range of tasks, particularly in computer vision. Early architectures like AlexNet [11] demonstrated the potential of deep learning for visual feature extraction, establishing a strong foundation for image classification. Building on this, more advanced CNNs such as ResNet [27] introduced deeper, more efficient designs that alleviated issues like vanishing gradients and enabled the extraction of more complex features. These improvements pushed the boundaries of performance in visual tasks, leading to substantial progress in object recognition and classification.

In the context of food recognition, CNN-based models have been widely adopted. However, as the need for handling more diverse, larger, and complex datasets arose, transformer-based architectures [28,29] began to gain popularity. By leveraging self-attention mechanisms, transformers excel in capturing long-range dependencies and contextual relationships, providing an advantage over CNNs in certain vision tasks. However, these architectures often come with higher computational costs and memory requirements, which limit their applicability in resource-constrained environments.

In response to these challenges, ConvNeXt [15] was introduced as an improved CNN framework inspired by transformer designs. ConvNeXt achieves superior performance over traditional CNNs and vision transformers while maintaining a more efficient computational profile. This makes it well suited for large-scale and real-time applications where both performance and efficiency are critical. Motivated by these advantages, our work adopts ConvNeXt as the backbone network, enabling high-quality feature extraction for food recognition tasks. This choice allows us to balance computational efficiency and performance, ensuring that the model can effectively handle the challenges posed by large-scale datasets while providing robust and accurate feature representations.

To further improve food recognition, we proposed and integrated several components into our framework, including the boundary-aware module (BAM), deformable ROI pooling (DRP), and a transformer encoder, followed by the NetRVLAD module. These components work synergistically to capture both global and local features, improving the model’s ability to address challenges such as intra-class variability, inter-class similarity, and domain shifts, which are commonly encountered in food recognition tasks. By combining a state-of-the-art backbone architecture with these specialized components, our approach pushes the boundaries of food recognition, achieving superior accuracy across multiple benchmark datasets of varying scales.

## 3. Materials and Methods

### 3.1. Overview of the Proposed Framework

**Figure 1:**
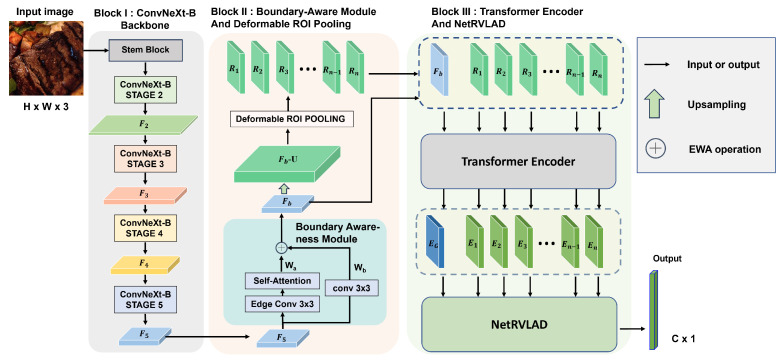
The proposed framework. The framework consists of three blocks, including the ConvNeXt-based backbone, the BAM and DRP, and the transformer encoder with the NetRVLAD module, for food recognition.

The proposed multi-stage DL framework, called CBDTN, is a CNN-based architecture consisting of three blocks: the ConvNeXt-based backbone for high-quality feature extraction, the BAM and DRP to refine the features and construct a global-local feature space, and the transformer encoder with the NetRVLAD module to model feature relationships and perform aggregation. As shown in Figure 1, Block I is the ConvNeXt-based backbone network, consisting of a stem block for processing input images and four stages that progressively extract hierarchical features, enabling robust and multi-scale feature representation. In Block II, the features extracted by the backbone network are first processed by the BAM to obtain boundary-sensitive global features. These features are then upsampled to generate feature maps with larger sizes. The upsampled feature maps are further processed using DRP to extract region-level local features with the same sizes as the boundary-sensitive global features. The global and local features are stacked to form a unified feature space, which serves as the input for Block III. In Block III, the input feature space is treated as a sequence and fed into a transformer encoder to model long-range relationships, producing encoded features. These encoded features are then passed to the NetRVLAD module, which performs clustering and soft assignment across different clusters to effectively aggregate diverse information within the feature space. This process enhances the utilization of convolutional features, improving the representational capacity of images within the same category while increasing the discriminative power of images across different categories. The final output, after passing through batch normalization (BN) and a softmax function, produces a feature vector of size C×1, where *C* represents the number of categories. This vector is used to represent the discriminative probabilities for each class, facilitating the classification of input images into their respective categories. The framework employs the cross-entropy loss function to optimize the classification performance, which is defined as

(1) $$L_{\mathrm{CE}}=-\frac{1}{N}\sum_{i=1}^{N}\sum_{j=1}^{C}y_{i,j}\log\left({\hat{y}}_{i,j}\right),$$

where *N* is the number of samples in a batch, *C* is the number of classes, $y_{i,j}$ is the ground-truth label for sample *i* and class *j*, and ${\hat{y}}_{i,j}$ is the predicted probability for the same.

### 3.2. Block I: The ConvNeXt Architecture

**Figure 2:**
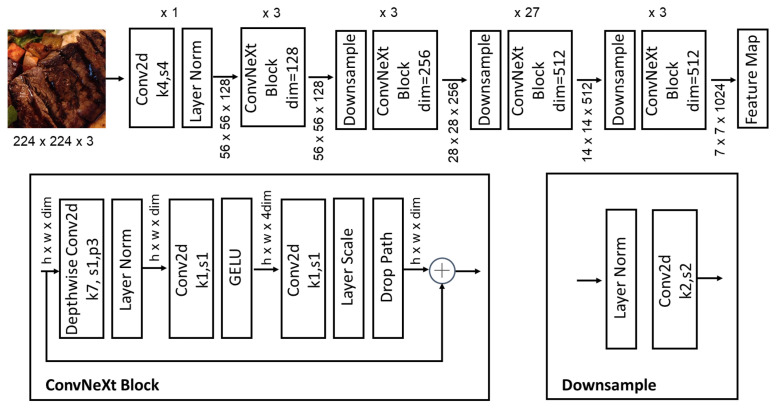
The detailed structure of the ConvNeXt-based network.

The proposed framework employs a ConvNeXt-based architecture [15] as its backbone, which is designed for efficient and hierarchical feature extraction, as depicted in Figure 1. The network begins with a stem block, which processes the input image to generate an initial feature map. The stem block utilizes a convolutional layer with a relatively large kernel size, followed by normalization (LayerNorm [30]) and a non-linear activation function (GELU [31]), capturing low-level spatial features while maintaining computational efficiency. Following the stem block, the backbone is divided into four hierarchical stages (Stage 2–Stage 5), each responsible for extracting increasingly abstract and semantically rich features. The transition between stages is facilitated by strided convolutions, which downsample the feature maps, effectively increasing the receptive field. The detailed structure of the ConvNeXt-based network is shown in Figure 2.

In each stage, the ConvNeXt-based architecture employs depthwise separable convolutions to enhance computational efficiency. This operation decomposes standard convolutions into depthwise and pointwise convolutions, significantly reducing the parameter count while preserving expressive power. For a given input tensor $X\in R^{H\times W\times C_{\mathrm{in}}}$, the depthwise convolution is expressed as

(2) $$Y=\mathrm{DepthwiseConv}(X,K_{d}),$$

where $K_{d}$ is the depthwise convolutional kernel, and the pointwise convolution refines the output channels to $C_{\mathrm{out}}$ through

(3) $$Z=\mathrm{PointwiseConv}(Y,K_{p}),$$

where $K_{p}$ is the pointwise kernel. The network incorporates residual connections, denoted as

(4) F(X)=X+T(X),

where T(X) represents the transformation function within each block. This design enhances gradient flow, mitigates vanishing gradients, and facilitates the training of deeper networks.

Feature maps are sequentially computed as $F_{2},F_{3},F_{4},$ and $F_{5}$ in Stages 2 through 5, respectively. Each stage increases the number of channels and computational depth, enabling the model to capture fine-grained and high-level semantic information. Specifically, the hierarchical structure ensures that lower stages extract local patterns, while higher stages focus on global context and object-level semantics. The final output, $F_{5}$, serves as a compact yet information-rich representation for downstream tasks.

### 3.3. Block II: The Boundary-Aware Module and Deformable ROI Pooling

The boundary-aware module is designed to enhance feature representation by explicitly incorporating boundary information and combining it with semantic features through a self-attention mechanism and weighted feature fusion. The module first extracts edge information using a Sobel filter-based convolution operator, which applies edge detection in the horizontal and vertical directions to emphasize boundary details. The edge features are subsequently passed through a 3×3 convolutional layer, followed by a self-attention mechanism. This mechanism takes the features as input and transforms them into query (*Q*), key (*K*), and value (*V*) matrices through learnable linear projections:

(5) $$Q=W_{q}F,\;K=W_{k}F,\;V=W_{v}F,$$

where *F* represents the input feature map and $W_{q},W_{k},W_{v}$ are the learnable weight matrices. The self-attention mechanism computes the attention weights by taking the scaled dot product of *Q* and *K*:

(6) $$A=\mathrm{softmax}\left(\frac{QK^{⊤}}{\sqrt{d_{k}}}\right),$$

where $d_{k}$ is the dimensionality of the key vectors. The output of the self-attention mechanism is then obtained by weighting *V* with the computed attention map:

(7) $$F_{\mathrm{attention}}=AV.$$

The self-attention output $F_{\mathrm{attention}}$ enhances the edge-aware feature representation by capturing long-range dependencies. This output is combined with semantic features extracted directly via a parallel 3×3 convolutional operation. The two feature maps are fused through an element-wise addition weighted by the learnable parameters $W_{a}$ and $W_{b}$:

(8) $$F_{b}=W_{a}\cdot F_{\mathrm{attention}}+W_{b}\cdot F_{\mathrm{semantic}},$$

where $F_{b}$ represents the boundary-aware feature map, $F_{\mathrm{attention}}$ denotes the enhanced features obtained via self-attention, and $F_{\mathrm{semantic}}$ corresponds to the semantic features.

**Figure 3:**
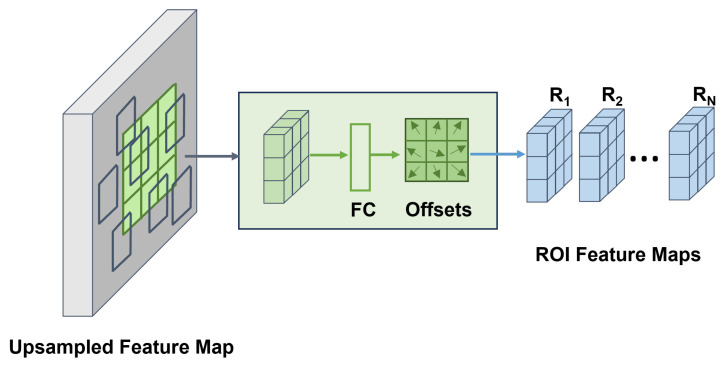
The structure of deformable ROI pooling module.

The boundary-aware feature map $F_{b}$ is then upsampled to a resolution of N×N, where *N* is a tunable hyperparameter. In our model, *N* is set to 42, ensuring that the upsampled feature map $F_{b-U}$ preserves both spatial resolution and boundary sensitivity. As shown in Figure 3, the upsampled feature map $F_{b-U}$ undergoes deformable ROI pooling, a process designed to adaptively aggregate features from regions of interest (ROIs) while accommodating geometric variations. Unlike standard ROI pooling, which applies fixed spatial bins, deformable ROI pooling predicts offsets for each bin based on the input feature map, thereby enhancing its ability to capture fine-grained and spatially misaligned features. For a given set of *n* ROIs, deformable ROI pooling produces feature maps, denoted as $\left\{R_{1},R_{2},\ldots ,R_{n}\right\}$, all with consistent dimensions.

The resulting ROI-specific features are combined with $F_{b}$ to form a unified global-local feature space, effectively integrating boundary-aware global features and region-specific local features. This composite feature space serves as the input to Block III, enabling robust downstream processing. The boundary-aware module thus provides a mechanism for capturing both contextual and boundary-sensitive details, crucial for tasks requiring precise spatial understanding.

### 3.4. Block III: The Transformer Encoder and the NetRVLAD Module

**Figure 4:**
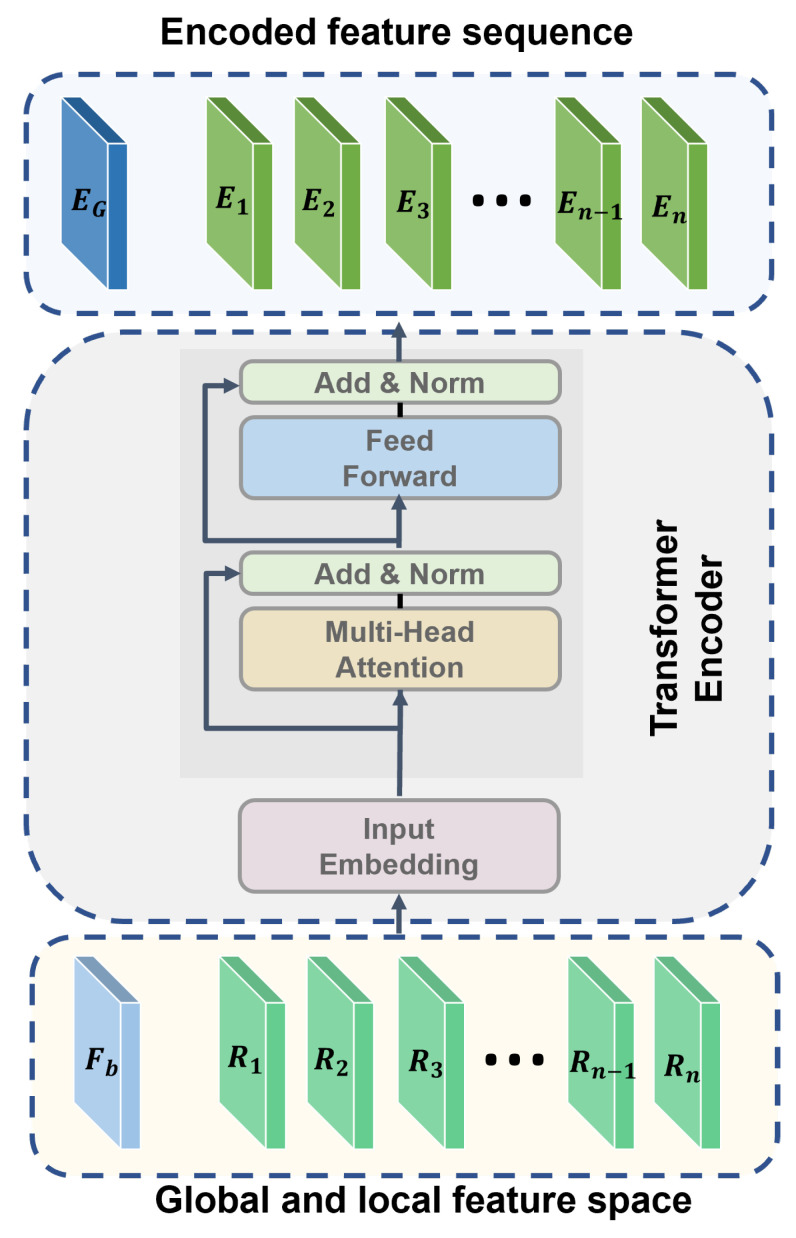
The structure of the deformable ROI pooling module.

The transformer encoder module is designed to encode both global and local features into a unified sequence representation, which is subsequently processed for classification. As shown in Figure 4, the input to this module consists of global features $F_{b}$ and region-specific local features $\left\{R_{1},R_{2},\ldots ,R_{n}\right\}$. These inputs are first projected onto a fixed-dimensional embedding space using a learnable linear projection layer:

(9) $$F_{\mathrm{proj}}=W_{\mathrm{embed}}F,\;F\in \left\{F_{b},R_{1},R_{2},\ldots ,R_{n}\right\},$$

where $W_{\mathrm{embed}}$ is the learnable weight matrix and $F_{\mathrm{proj}}$ represents the projected input embeddings. The sequence of embeddings is then passed through a transformer encoder block. Each encoder block consists of a multi-head self-attention mechanism, followed by layer normalization and a position-wise feed-forward network.

The multi-head self-attention mechanism calculates the attention weights by projecting the input embeddings onto query (*Q*), key (*K*), and value (*V*) matrices:

(10) $$Q=W_{q}F_{\mathrm{proj}},\;K=W_{k}F_{\mathrm{proj}},\;V=W_{v}F_{\mathrm{proj}},$$

where $W_{q},W_{k},W_{v}$ are the learnable projection matrices. The attention output is computed as

(11) $$A=\mathrm{softmax}\left(\frac{QK^{⊤}}{\sqrt{d_{k}}}\right)V,$$

where $d_{k}$ is the dimensionality of the key vectors. The attention output is subjected to dropout and added back to the input via a residual connection, followed by layer normalization:

(12) $$F_{\mathrm{att}}=\mathrm{LayerNorm}\left(A+F_{\mathrm{proj}}\right).$$

Next, the normalized attention output is passed through a feed-forward neural network (FFNN) consisting of two dense layers with ReLU activation:

(13) $$F_{\mathrm{FFN}}=\mathrm{ReLU}\left(W_{1}F_{\mathrm{att}}+b_{1}\right)W_{2}+b_{2},$$

where $W_{1},W_{2}$ and $b_{1},b_{2}$ are learnable parameters. A second residual connection and layer normalization are applied to produce the final encoded features:

(14) $$F_{\mathrm{encoded}}=\mathrm{LayerNorm}\left(F_{\mathrm{FFN}}+F_{\mathrm{att}}\right).$$

The output of the encoder is a sequence of encoded features $\left\{E_{g},E_{1},E_{2},\ldots ,E_{n}\right\}$, where $E_{g}$ represents the global feature embedding and $\left\{E_{1},E_{2},\ldots ,E_{n}\right\}$ corresponds to the region-specific features.

The NetRVLAD module processes a sequence of encoded features $\left\{E_{g},E_{1},E_{2},\ldots ,E_{n}\right\}$, where $E_{g}$ represents the globally encoded feature vector and $E_{1},E_{2},\ldots ,E_{n}$ corresponds to locally encoded features derived from individual ROIs. The module aggregates these feature descriptors into a compact and discriminative global representation suitable for classification tasks.

Each feature vector $E_{i}\in R^{\mathrm{feature\_size}}$ (where i∈{g,1,…,n}) is first projected onto a cluster-assignment space using a trainable weight matrix $W_{\mathrm{cluster}}\in R^{\mathrm{feature\_size}\times \mathrm{cluster\_size}}$ and a bias vector $b_{\mathrm{cluster}}\in R^{\mathrm{cluster\_size}}$. For a given feature vector $E_{i}$, the cluster activation scores are computed as

(15) $$A_{i}=\mathrm{softmax}\left(E_{i}W_{\mathrm{cluster}}+b_{\mathrm{cluster}}\right),$$

where $A_{i}\in R^{\mathrm{cluster\_size}}$ represents the soft assignment of $E_{i}$ to the predefined clusters. The softmax function ensures that each feature is probabilistically distributed across all clusters.

Next, all input feature vectors are aggregated by weighting them according to their cluster assignments. For the entire sequence of encoded features, the cluster-wise aggregated features are computed as

(16) $$V_{c}=\sum_{i=1}^{n+1}A_{i,c}E_{i},$$

where $A_{i,c}$ is the *c*-th cluster assignment score for the *i*-th feature vector and $V_{c}\in R^{\mathrm{feature\_size}}$ represents the aggregated feature descriptor for the *c*-th cluster. This process results in a VLAD representation $V\in R^{\mathrm{cluster\_size}\times \mathrm{feature\_size}}$, where each row corresponds to the aggregated descriptor of a cluster.

The VLAD representation *V* is normalized along the feature dimension using L2 normalization to ensure robustness to scale variations:

(17) $$\hat{V}=L2\mathrm{Norm}\left(V\right).$$

Finally, the normalized VLAD representation $\hat{V}$ is flattened into a vector of dimension cluster_size · feature_size and projected onto the output space of dimension output_dim using a trainable matrix $W_{N}\in R^{(\mathrm{cluster\_size}\cdot \mathrm{feature\_size})\;\times \;\mathrm{output\_dim}}$:

(18) $$\hat{y}=\hat{V}W_{N},$$

where $\hat{y}\in R^{\mathrm{output\_dim}}$ is the final compact feature representation.

In this framework, the global feature $E_{g}$ and local features $\left\{E_{1},\ldots ,E_{n}\right\}$ are effectively aggregated into a unified global-local feature space. The resulting representation captures both global context and local discriminative details, enabling accurate classification when passed through subsequent normalization (BatchNorm) and classification layers (softmax).

This combination of a transformer encoder and NetRVLAD module enables effective modeling of both global and local contexts while ensuring robust feature clustering and aggregation, which are critical for high-performance classification tasks.

## 4. Results

### 4.1. Datasets and Training Settings

To evaluate the performance of the proposed model, we conducted experiments on three widely used public datasets for food recognition: EPFL Food-5k [32], ETHZ Food-101 [12], and Food-2k [18]. EPFL Food-5k comprises 5000 images, evenly divided into 2500 food-related and 2500 non-food images. The images were sourced from wearable cameras and mobile phones, providing a high degree of variability in content and quality. The dataset is split into 3000 images for training, 1000 for validation, and 1000 for testing. ETHZ Food-101 is the first large-scale dataset for Western cuisine recognition, containing a total of 101,000 images across 101 food categories. The dataset is officially divided into 75,750 images for training and validation, and 25,250 images for testing, ensuring a standardized benchmark for evaluation. Food-2k, released in 2023, is the largest food recognition dataset, consisting of 1,036,564 images spanning 2000 food categories. The dataset is partitioned into training (60%), validation (10%), and testing (30%) subsets, offering extensive coverage of diverse food types and enabling robust model training and evaluation. The distributions and details of the three datasets are summarized in Table 1.

We trained our framework with an input image size of 224 × 224, a batch size of 8, and the SGD [33] optimizer on eight NVIDIA RTX A6000 GPUs using the Keras framework for 200 epochs. The initial learning rate was set to 0.0001 and was adjusted dynamically during training to optimize convergence. Specifically, the learning rate was reduced by a factor of 10 every 50 epochs. During training, images were resized to 256 × 256 and then augmented with random rotations ($\pm 15^{\circ }$), scaling (±15%), horizontal flipping (50% probability), and random cropping to match the target input size, which enhances robustness by simulating real-world variations. The FLOPs for the proposed CBDTN model were measured at 1.22 G, and the number of trainable parameters was 591.19 M.

### 4.2. Evaluation Metrics

To achieve a comprehensive assessment, multiple and contrasting evaluation metrics were employed. These metrics included sensitivity (Sen), precision (Pre), and specificity (Spe), accuracy (Acc) and are defined below.

Sensitivity measures the proportion of true positives correctly identified by the model:

(19) $$\mathrm{Sensitivity}=\frac{TP}{TP+FN}$$

where TP is the number of true positives and FN is the number of false negatives.

Precision indicates the proportion of predicted positives that are true positives:

(20) $$\mathrm{Precision}=\frac{TP}{TP+FP}$$

where FP is the number of false positives.

Specificity measures the proportion of true negatives correctly identified:

(21) $$\mathrm{Specificity}=\frac{TN}{TN+FP}$$

where TN is the number of true negatives.

Accuracy represents the overall correctness of the model’s predictions:

(22) $$\mathrm{Accuracy}=\frac{TP+TN}{TP+TN+FP+FN}$$

where TP, TN, FP, and FN are defined as above.

### 4.3. Experimental Results

To comprehensively evaluate the effectiveness of the proposed method, we conducted comparative experiments on the three datasets against several state-of-the-art approaches across multiple evaluation metrics, including Acc, Sen, Pre, and Spe. The results demonstrate the superiority of our method, achieving consistent improvements across these metrics. Additionally, to further validate the contribution of each component in the proposed framework, we performed ablation studies. These ablation experiments systematically examined the impact of each module, providing insights into their specific roles in enhancing overall model performance.

#### 4.3.1. Comparative Results on the EPFL Food-5k Dataset

The EPFL Food-5k dataset comprises only two categories: food and non-food. To comprehensively evaluate model performance, we calculated the true positives (TP), true negatives (TN), false positives (FP), and false negatives (FN) based on the predictions made on the test set. We conducted comparative experiments on the EPFL Food-5k dataset, benchmarking our approach against several state-of-the-art methods, including GoogLeNet [32], ResNet-152+SVM [34], KenyanFC [35], and VGG16 [36]. The results summarized in Table 1 highlight the significant advantages of our method across all evaluation metrics: sensitivity (Sen), precision (Pre), specificity (Spe), and accuracy (Acc), providing a detailed assessment of the model’s capability.

As shown in Table 2, among the compared methods, ResNet-152+SVM exhibited the best performance, achieving a high accuracy of 0.9940, along with sensitivity and specificity scores of 0.9960 and 0.9920, respectively. KenyanFC and GoogLeNet also performed well, both with accuracies of 0.9920, but they showed slightly lower precision and specificity compared to ResNet-152+SVM. VGG16 delivered the lowest overall performance among the baselines, with an accuracy of 0.9900.

The proposed method outperformed all compared approaches, achieving the highest accuracy (0.9980), along with significant improvements in precision (0.9960) and specificity (0.9960). Furthermore, our model maintained a perfect sensitivity score (1.0000), highlighting its exceptional ability to identify positive instances. These results indicate that the proposed method not only achieves superior classification accuracy but also balances the trade-offs between identifying positive and negative samples, as reflected in the metrics. The results emphasize the robustness of our method and its ability to address challenges in food image classification, surpassing existing state-of-the-art approaches on the EPFL Food-5k dataset.

#### 4.3.2. Comparative Results on the ETHZ Food-101 Dataset

To further demonstrate the effectiveness of our proposed method, we conducted experiments on the ETHZ Food-101 dataset, a widely used benchmark in the food recognition domain. As is standard in this field, the Top-1 accuracy (Acc) was employed as the primary evaluation metric to ensure a fair and direct comparison with existing methods. Additionally, some studies in the domain also report the Top-5 accuracy (Top-5Acc) as a complementary, more lenient metric to provide further insights into model performance.

Reporting both the Top-1 accuracy and Top-5 accuracy provides a more holistic evaluation of model performance. While the Top-1 accuracy reflects the strict classification capability of the model, the Top-5 accuracy offers insight into the model’s ability to identify the correct class among the top candidates, which is especially useful in scenarios with high intra-class variability or subtle inter-class differences. The comparative results are presented in Table 3.

The proposed method achieved a Top-1 accuracy of 0.9917, significantly outperforming all competing methods. Among the existing approaches, ViT [25] demonstrated strong performance, achieving an accuracy of 0.9517, followed by ConvNeXt-AG [16] with 0.9112 and LP-ViT [26] with 0.9070. CNN54 [14], a deeper convolutional neural network, attained 0.8828, while EfficientNet-b0 [13] and AlexNet [12] showed relatively lower accuracies of 0.8000 and 0.5640, respectively.

The remarkable improvement in accuracy by our method highlights its superior capability in addressing the challenges of food recognition, such as high intra-class variance and inter-class similarity. By using the standard Top-1 accuracy metric, this evaluation ensures comparability with prior methods and underscores the robustness and generalizability of the proposed framework in the context of large-scale food recognition tasks.

#### 4.3.3. Comparative Results on the Food-2k Dataset

We conducted comparative experiments on the Food-2k dataset, the largest and most challenging benchmark in the food recognition domain. Both the Top-1 accuracy (Top-1Acc) and Top-5 accuracy (Top-5Acc) were used as evaluation metrics to ensure a comprehensive assessment of the model’s classification performance. The results summarized in Table 4 highlight the advantages of our approach compared to several state-of-the-art methods.

The proposed method achieved the highest performance in both the Top-1 accuracy (0.8587) and Top-5 accuracy (0.9828), outperforming all competing methods. Among the baseline methods, PRENet(ResNet101) [18] delivered the best performance after ours, with a Top-1 accuracy of 0.8375 and a Top-5 accuracy of 0.9733, closely followed by SENet154 [37], with a Top-1 accuracy of 0.8362 and a Top-5 accuracy of 0.9722. MSPTN [38] also showed competitive results, achieving a Top-1 accuracy of 0.8288 and a Top-5 accuracy of 0.9712. In contrast, methods such as PMG [39], MOMN [40], and ViT-CNN [41] demonstrated relatively lower performance, with Top-1 accuracies ranging from 0.8079 to 0.8129 and Top-5 accuracies ranging from 0.9574 to 0.9612.

The notable increase in the Top-5 accuracy (Top-5Acc=0.9828) also highlights the model’s capability to rank the true class consistently among the top predictions, an important characteristic for applications requiring flexible decision making. These results affirm the superiority of the proposed framework over state-of-the-art methods on the Food-2k dataset.

**Table 4:** Comparative results on the Food-2k dataset.

Method	Top-1 Acc	Top-5 Acc
PMG [39]	0.8129	0.9612
MOMN [40]	0.8084	0.9602
SENet154 [37]	0.8362	0.9722
ViT-CNN [41]	0.8079	0.9574
MSPTN [38]	0.8288	0.9712
PRENet(ResNet101) [18]	0.8375	0.9733
CBDTN (Ours)	**0.8587**	**0.9828**

#### 4.3.4. Ablation Study

To analyze the contribution of each module in the proposed framework, we conducted an ablation study on the three datasets: Food-5k, Food-101, and Food-2k. The results are summarized in Table 5, demonstrating the impact of removing specific components on overall performance.

Effect of BAM (Boundary-Aware Module): Removing the BAM (denoted as w/o BAM) resulted in a significant drop in accuracy across all datasets, with reductions of 0.6% on Food-5k, 0.9% on Food-101, and 0.75% on Food-2k. This highlights the importance of boundary-sensitive features in capturing fine-grained details for improved classification accuracy.

Effect of DRP (Deformable ROI Pooling): When replacing DRP with a simpler approach of directly repeating and stacking boundary-sensitive features (w/o DRP), the performance further decreased, with accuracy on Food-5k dropping to 0.9900, accuracy on Food-101 dropping to 0.9400, and accuracy on Food-2k dropping to 0.8228. These results underscore the ability of DRP to enhance feature localization and capture regional variations effectively.

Effect of Transformer Encoder: Substituting the transformer encoder with a fully connected (FC) layer for feature dimension processing (w/o transformer encoder) also led to a considerable performance reduction. The accuracy dropped by 0.8% on Food-5k, 1.7% on Food-101, and 2.62% on Food-2k, emphasizing the importance of the transformer encoder in capturing global and local dependencies in the feature space.

Effect of NetRVLAD Module: Replacing the NetRVLAD module with a simpler FC layer to directly output class probabilities (w/o NetRVLAD) reduced the accuracy to 0.9940, 0.9846, and 0.8356 on Food-5k, Food-101, and Food-2k, respectively. These results demonstrate the effectiveness of the NetRVLAD module in clustering and aggregating discriminative features for robust classification.

Overall Impact: The complete framework (CBDTN) consistently achieved the best performance across all datasets, with accuracies of 0.9980, 0.9917, and 0.8587 on Food-5k, Food-101, and Food-2k, respectively. The ablation results confirm the critical contributions of the BAM, DRP, transformer encoder, and NetRVLAD module to the proposed framework, each playing a distinct and complementary role in enhancing classification accuracy.

## 5. Discussion

In this study, we addressed key challenges in food recognition, including variability in food presentation, intra-class diversity, and inter-class similarity. These challenges arise due to the diverse nature of food items, such as varying preparation styles, overlapping appearances, and the differing contexts in which food is photographed. To overcome these issues, we proposed a novel framework that integrates a boundary-aware module (BAM), deformable ROI pooling (DRP), a transformer encoder, and a NetRVLAD module. These components were designed to jointly enhance the model’s capability for robust feature extraction, fine-grained discrimination, and effective aggregation of global and local features.

The proposed BAM effectively captures boundary-sensitive features, aiding in the differentiation of highly similar food items. DRP further refines these features by localizing critical regions, enabling adaptive representation of subtle intra-class differences. The transformer encoder integrates global dependencies and contextual relationships, which are crucial for distinguishing visually similar classes. The NetRVLAD module provides robust feature clustering and aggregation, ensuring the extraction of discriminative embeddings for final classification.

Quantitative results across three diverse datasets, Food-5k, Food-101, and Food-2k, demonstrate the superiority of the proposed method. The framework consistently achieved state-of-the-art performance across multiple evaluation metrics, with significant improvements in accuracy compared to existing methods. For instance, on the challenging Food-101 dataset, our framework outperformed the previously best method by a notable margin, achieving a Top-1 accuracy of 99.17%. Ablation studies further validated the contributions of each module, showing complementary and synergistic effects when integrated into the complete framework.

The proposed method holds significant promise for advancing intelligent dietary management systems. Its superior performance in food recognition could be instrumental in applications such as automated meal tracking, calorie estimation, and personalized nutrition planning. By accurately identifying and classifying food items in diverse scenarios, our framework can provide reliable and efficient solutions to support healthy eating habits and dietary interventions.

Despite its outstanding performance, the current model also has limitations. Specifically, the framework’s reliance on high-capacity components, such as transformer encoders, requires substantial computational resources and memory, posing challenges for deployment on resource-constrained devices. To address this, future work will focus on making the model more lightweight to enhance its applicability in mobile and embedded systems. Techniques such as knowledge distillation, model pruning, and quantization will be explored to achieve a balance between performance and computational efficiency.

Current methods mainly focus on food recognition and analysis using single images. However, exploring dynamic food recognition based on video data and incorporating multi-modal data (e.g., nutritional information) for comprehensive nutritional analysis remain open challenges. Addressing these aspects will further enhance the framework’s practical applicability in real-world scenarios, enabling more robust and versatile solutions for real-time meal tracking and personalized nutrition.

Overall, the proposed framework’s potential extends beyond its current application in food recognition. It may serve as a foundational tool for broader dietary management systems, paving the way for accessible and efficient solutions to support global health initiatives. By continuing to refine and adapt the model, we aim to advance its utility in real-world scenarios, further contributing to the development of smart dietary management tools.

## 6. Conclusions

In conclusion, this study proposed a multi-stage DL framework for food recognition, addressing critical challenges such as high intra-class diversity and inter-class similarity in food datasets. The framework integrates a boundary-aware module (BAM) for boundary-sensitive feature extraction, deformable ROI pooling (DRP) for localized feature refinement, a transformer encoder for capturing global contextual relationships, and a NetRVLAD module for robust feature aggregation. These components work synergistically to enhance the model’s ability to handle complex visual variations in food items.

The framework was evaluated comprehensively on three benchmark datasets: Food-5k, Food-101, and Food-2k, consistently outperforming state-of-the-art methods across multiple evaluation metrics. On the Food-101 dataset, the proposed model achieved a Top-1 accuracy of 99.17%, surpassing the previously best existing method by a notable margin. Similarly, on the Food-2k dataset, the model achieved a Top-1 accuracy of 85.87% and a Top-5 accuracy of 98.28%, demonstrating its robustness and generalizability. Ablation studies further validated the contributions of individual modules, showcasing the complementary effects of the proposed architectural innovations.

The outstanding performance highlights the framework’s potential as a foundational tool for intelligent dietary management systems, enabling applications such as automated meal tracking, calorie estimation, and personalized nutrition planning. By delivering high accuracy and robustness, the proposed method addresses real-world challenges in food recognition, paving the way for smarter and more efficient solutions in dietary management.

## Figures and Tables

**Table 1:** The distributions and details of the EPFL Food-5k, ETHZ Food-101, and Food-2k datasets.

Dataset	Categories	Train-Val	Test	Total
EPFL Food-5k	2	4000	1000	5000
ETHZ Food-101	101	75,750	25,250	101,000
Food-2k	2000	724,705	311,859	1,036,564

**Table 2:** Comparative results on the EPFL Food-5k dataset.

Method	Sen	Pre	Spe	Acc
GoogLeNet [32]	0.9960	0.9880	0.9880	0.9920
ResNet-152+SVM [34]	0.9960	0.9930	0.9920	0.9940
KenyanFC [35]	0.9940	0.9900	0.9890	0.9920
VGG16 [36]	0.9920	0.9880	0.9880	0.9900
CBDTN (ours)	**1.0000**	**0.9960**	**0.9960**	**0.9980**

**Table 3:** Comparative results on the ETHZ Food-101 dataset.

Method	Top-1 Acc	Top-5 Acc
AlexNet [12]	0.5640	-
EfficientNet-b0 [13]	0.8000	-
CNN54 [14]	0.8828	0.9688
ConvNeXt-AG [16]	0.9112	0.9877
ViT [25]	0.9517	-
LP–ViT [26]	0.9070	-
CBDTN (Ours)	**0.9917**	**0.9996**

**Table 5:** Ablation study results.

Method	Acc (Food-5k)	Acc (Food-101)	Acc (Food-2k)
CBDTN	0.9980	0.9917	0.8587
w/o BAM	0.9920	0.9827	0.8512
w/o DRP	0.9900	0.9400	0.8228
w/o transformer encoder	0.9900	0.9747	0.8325
w/o NetRVLAD	0.9940	0.9846	0.8356

w/o means “without”, indicating the exclusion of specific model components in the experiment.

## Data Availability

The data supporting the findings of this study are publicly available [Food-5k, Food-101, Food-2k]. The datasets can be accessed at the following links: https://www.kaggle.com/datasets/trolukovich/food5k-image-dataset (accessed on on 22 January 2025), https://data.vision.ee.ethz.ch/cvl/datasets_extra/food-101/ (accessed on 22 January 2025), http://123.57.42.89/FoodProject.html (accessed on on 22 January 2025). Access to these datasets requires registration and approval from the dataset provider.
